# Genome-wide association study Identified multiple Genetic Loci on Chilling Resistance During Germination in Maize

**DOI:** 10.1038/s41598-017-11318-6

**Published:** 2017-09-07

**Authors:** Guanghui Hu, Zhao Li, Yuncai Lu, Chunxia Li, Shichen Gong, Shuqin Yan, Guoliang Li, Mingquan Wang, Honglei Ren, Haitao Guan, Zhengwei Zhang, Dongling Qin, Mengzhu Chai, Juping Yu, Yu Li, Deguang Yang, Tianyu Wang, Zhiwu Zhang

**Affiliations:** 1 College of Agriculture, Northeast Agricultural University, Harbin, 150030 Heilongjiang China; 2 Institute of Maize Research, Heilongjiang Academy of Agricultural Sciences, Harbin, 150086 Heilongjiang China; 3 Department of Crop and Soil Sciences, Washington State University, Pullman, 99164 WA USA; 4 College of Agricultural Resources and Environment, Heilongjiang University, Harbin, 150001 Heilongjiang China; 5 Quality & Safety Inst. of Agricultural Products, Heilongjiang Academy of Agricultural Sciences, Harbin, 150086 Heilongjiang China; 6 Institute of Crop Science, Chinese Academy of Agricultural Sciences, Beijing, 100081 China

## Abstract

Maize (*Zea mays*, L.) cultivation has expanded greatly from tropical to temperate zones; however, its sensitivity to chilling often results in decreased germination rates, weak seedlings with reduced survival rates, and eventually lower yields. We conducted germination tests on the maize-282-diverse-panel (282 inbred lines) under normal (25 °C) and chilling (8 °C) conditions. Three raw measurements of germination were recorded under each condition: 1) germination rate, 2) days to 50% germination, and 3) germination index. Three relative traits were derived as indicators of cold-tolerance. By using the 2,271,584 single nucleotide polymorphisms (SNPs) on the panel from previous studies, and genome-wide association studies by using FarmCPU R package to identify 17 genetic loci associated with cold tolerance. Seven associated SNPs hit directly on candidate genes; four SNPs were in high linkage disequilibrium with candidate genes within 366 kb. In total, 18 candidate genes were identified, including 10 candidate genes supported by previous QTL studies and five genes supported by previous gene cloning studies in maize, rice, and Arabidopsis. Three new candidate genes revealed by two associated SNPs were supported by both QTL analyses and gene cloning studies. These candidate genes and associated SNPs provide valuable resources for future studies to develop cold-tolerant maize varieties.

## Introduction

Worldwide, more maize (*Zea mays, L*.) is produced (tons) than any other cereal crop, providing a critically important source of food for people, feed for livestock, and biofuel for energy. As world population continues to increase, maintaining the current supply and finding ways to increase the future production of maize are imperative. One strategy has been to simply expand the geographic area planted to maize. Indeed, during the last century, cultivation of maize has expanded into mountainous regions and higher latitudes, even as far as 57°N^1^. However, because maize originated from tropical regions, it is inherently sensitive to low temperatures. When cultivated under lower temperatures, maize experiences shorter growing seasons, slower growth rates, and higher seedling vitality, all of which result in variable or reduced yields^2^.

Because of fluctuating weather conditions due to changing environments, cold temperatures always present production risks. Extending the growth period by planting maize earlier in the spring in higher-latitude or mountainous regions could increase productivity because a longer growth period results in more biomass accumulation^3^. However, the success of early planting will require higher seed vigor, faster germination times, and better germination rates under cold temperature conditions.

Chilling temperatures, ranging from 0 to 15 °C, cause changes in maize morphology and physiology during early growth stages. Morphologically, chilling prolongs germination time and growth duration, reduces germination rate, and weakens young seedlings^4^. Physiologically, chilling initiates a series of processes, including detrimental changes in salicylic acid (SA)^5^, glycinebetain (GB)^6^, antioxidant defense^7^, Ca^2+^ influx, membrane fluidity^8^, metabolism^9^, and the photosynthetic apparatus^10^. The evidence links Cold-Regulated (COR) gene expression with changes in many of these morphological and physiological traits, indicating that genetic breeding may be a successful strategy for enhancing cold tolerance in maize^2^.

Quantitative trait loci (QTL), COR gene expression and protein activities associated with cold-related traits in maize, have been investigated and partially identified by QTL linkage mapping^11–15^. For instance, at suboptimal temperatures, QTLs significantly associated with Malic Enzyme (ME) content were detected on chromosomes 1 and 3^16^. Other QTLs, detected on chromosomes 2 and 3, were significantly associated with ascorbate (Vitamin C) and chlorophyll “a” content, respectively. Three QTLs, significantly associated with photosynthetic efficiency (Fv/Fm), were mapped on chromosomes 3, 4, and 8 under chilling-dependent and photo inhibition conditions^15^. A major QTL on chromosome 6 was linked to the cold-tolerance of photosynthesis under chilling conditions (15/13 °C) (day/night). And, in two different maize populations, a QTL on chromosome 2 was significantly associated with the cold-tolerance of photosynthesis^17^. However, these findings are less sensitive due to the genetic limitations imposed by using bi-parental mapping populations. This limitation produces low resolution on QTL mapping because of the long linkage disequilibrium extent^18^.

The development of high-throughput DNA sequencing technologies, including single nucleotide polymorphism (SNP) genotyping and genotyping-by-sequencing (GBS), allows rapid and economical genotyping for many individuals. Genome-Wide Association Study (GWAS) mapping became a powerful alternative, based on linkage disequilibrium (LD) and sufficient genetic background information provided by diverse maize panels^19^. This approach has been successfully used to map QTLs at a better resolution and to detect candidate genes associated with diseases in humans, animals, and plants^20^. GWAS has also been used to detect a few significant SNPs associated with cold-tolerant traits at the seed germination and seedling stages in maize^21–23^. For example, the SNPs associated with relative germination rate (based on bud emergence) under chilling conditions have been detected on chromosomes 1 (position: 146384303), 2 (position: 79181517), 4 (position: 148401277), and 7 (position: 51743082)^23^. Also, a SNP associated with relative root number at germination was found on chromosome 2 (position:89269445)^23^ and a SNP associated with Fv/Fm was located on chromosome 1 (position:248397011)^22^.

Although some potential cold-tolerant genes and QTLs have been identified in maize, the genetic studies of chilling stress on germination is limited. In this study, we selected a diverse maize population panel, composed of 282 inbred lines, that has been successfully used to investigate quantitative traits via GWAS^24^. Our study was designed to accomplish the following objectives: 1) perform GWAS to identify potential SNPs responsible for chilling tolerance during development of root radicles, 2) compare our GWAS results with previous QTL mapping results, and 3) identify and evaluate candidate genes for future studies.

## Results

### Germination performance

The diverse maize association panel demonstrated much greater variation in germination rates (GRs) on day 21 under chilling conditions (8 °C) compared to the panel’s GRs on day 7 under normal conditions (25 °C). Overall, seed GR was substantially suppressed under chilling conditions (median = 30.0%) in contrast to normal conditions (median = 90.00%) — even when GR was measured as far out as the 21^st^ day under chilling (Table 1).

**Table 1:** Descriptive statistics of the germination traits under chilling and normal conditions*.

Trait	n	Mean	Median	SD	Range
GR21_C (%)	241	41.48	30.00	37.48	0.00~100.00
GR_N (%)	241	84.89	90.00	15.56	50.00~100.00
RGR	241	0.46	0.35	0.39	0.00~1
DT50_C (day)	241	22.67	25.67	7.63	10.00~31.00
DT50_N (day)	241	2.77	2.67	0.76	1.00~7.00
RDT50	241	8.57	8.45	3.53	2.94~29.00
GI_C	241	0.64	0.37	0.62	0.00~2.00
GI_N	241	1.72	1.75	0.37	0.42~2.46
RGI	241	0.37	0.24	0.39	0.00~2.63

^*^Statistics include number of observations (n), mean, median, standard deviation (SD), and range. The maize-282-diverse-panel was evaluated for 241 inbred lines, with enough seeds for germination tests under optimum (25 °C), or Normal (“_N”) conditions, and Chilling (“_C”) conditions (8 °C). Directly observed traits included Germination Rate (GR) on the 21^st^ day under chilling conditions (GR21_C) and on the 7^th^ day under normal conditions (GR_N); Days To 50% germination under Chilling (DT50_C) and Normal conditions (DT50_N); and Germination Index under Chilling (GI_C) and Normal conditions (GI_N) as GI = ∑(Gt/Tt), where Gt equals the number of seeds newly germinated on day t and Tt equals the number of days elapsed. In addition, three Relative (R) traits were derived by dividing the directly measured trait values (GR, DT50, and GI) under chilling conditions by their corresponding values under normal conditions. In total, we evaluated nine traits, described in detail as follows:

GR21_C: Germination (root emergence) rate at 21 days under chilling conditions.

GR_N: Germination (root emergence) rate at 7 days under normal (control) conditions.

RGR: Relative germination rate (GR21_C/GR_N).

DT50_C: Days to 50% root germination under chilling conditions (up to day 31).

DT50_N: Days to 50% root germination under normal (control) conditions.

RDT50: Relative days to 50% root germination (DT50_C/DT50_N).

GI_C: Germination index from 0 to 31 days under chilling conditions.

GI_N: Germination index from 0 to 7 days under normal (control) conditions.

RGI: Relative germination index (GI_C/GI_N).

**Figure 1:**
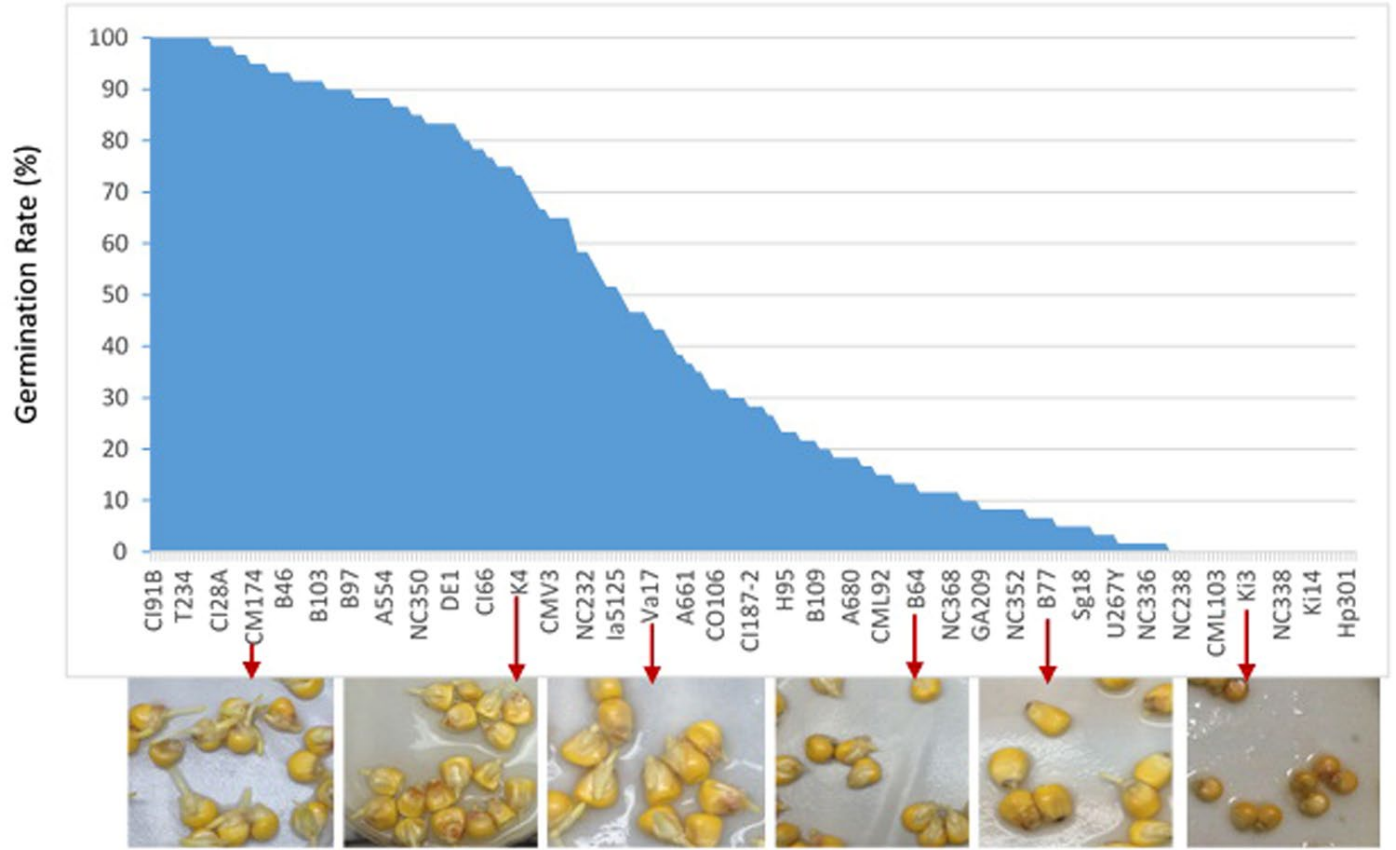
Seed germination of the diverse maize panel under chilling conditions. Germination was defined as root emergence from seed. The chilling condition was set to a temperature of 8 °C. Root emergence was observed daily for 31 days. Germination rates were calculated on the 21^st^ day for all 241 inbred lines. Above, these inbred lines are sorted based on their germination rates, from high (left) to low (right). Several of the line names are displayed across the horizontal axis. Six lines are selected (red arrows) to demonstrate their degree of root emergence with pictures.

We also found extreme differences in GR among the 241 inbred lines of the diverse maize association panel under chilling conditions (Fig. 1). For example, 14 lines (A632, B37, B57, C49A, CI91B, CM37, Co255, EP1, NC310, Oh43E, R109B, R4, T232, and T234) exhibited 100% germination rates and 48 lines exhibited germination rates above 85%. At the other extreme, 36 lines experienced no germination before the 21^st^ day; however, we did observe germinations in these lines after the 21^st^ day.

Although day 21 has been used as the standard time to measure germination rate under chilling conditions, extending the observations and measuring additional traits are critically needed to adequately monitor germination rate. One of the additional traits we measured was days to 50% germination (DT50). To obtain robust measurements of DT50, we continued observing germination for a total of 31 days. The accumulative measurements indicated that the initial and final values of DT50 were 10.3 and 29.6 days, respectively, using the Boltzmann function (Supplementary Table 1 and Supplementary Figure 1), which fit the observed DT50 well (adjusted R square = 99.56%). This result suggested that the 31-day period was sufficient to cover the observations. All but 37 lines reached 50% germination by the end of 31 days. For these 37 lines, we adjusted the observations back to 31 days using the method developed to handle outlier gene expressions from 5,000 maize inbred lines^25^.

Another additional trait we measured was germination index (GI), which was detailed in the Materials and Methods section. For GR, DT50 and GI, we derived corresponding relative traits by dividing each trait’s chilling condition value by its normal condition value, allowing us to make valid comparisons between the two conditions. The relative GR (RGR) had a median of 0.40 (Table 1). The relative DT50 (RDT50) had a median of 8.45, which means that maize seedlings grown under chilling conditions took eight-fold longer to reach 50% germination than when grown under normal conditions.

**Figure 2:**
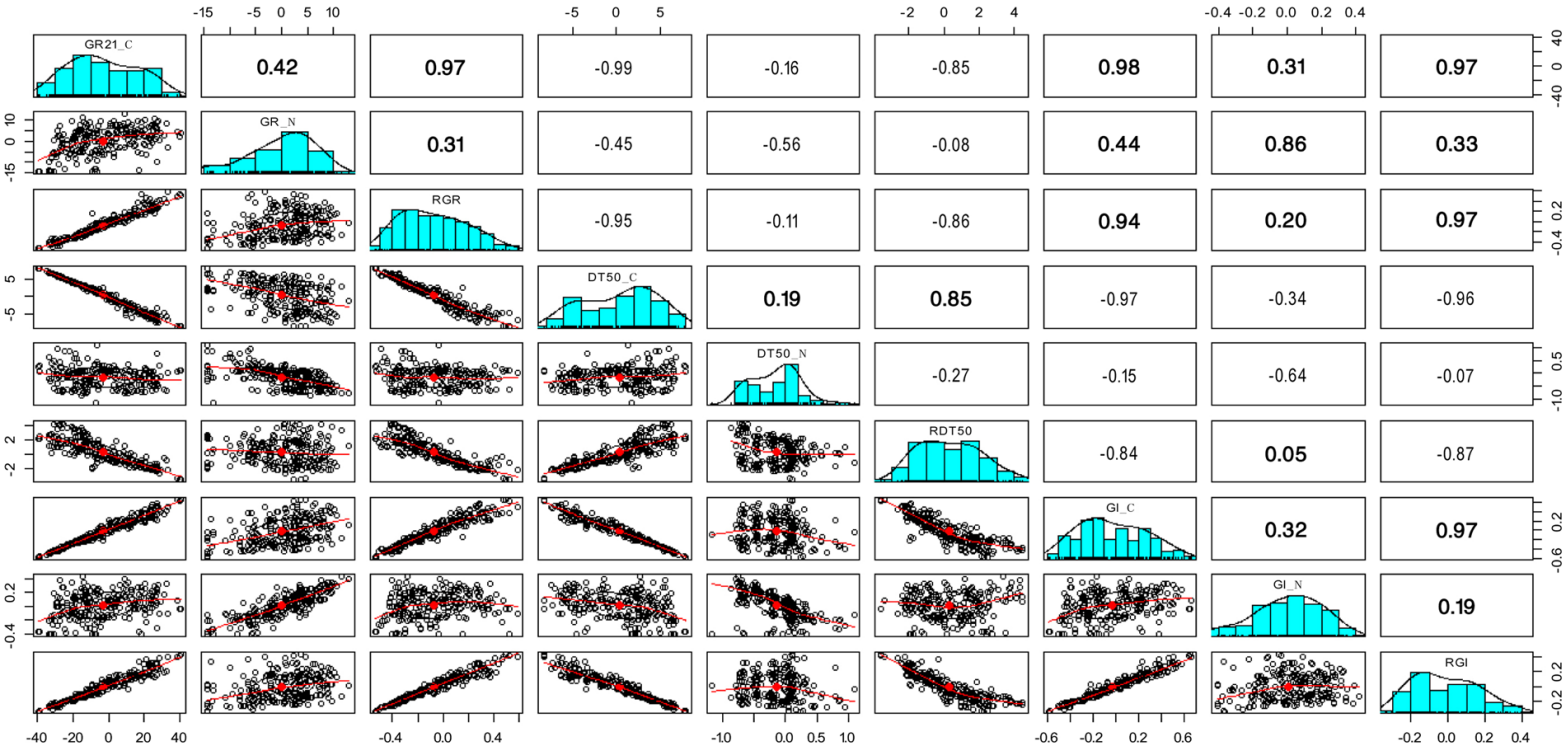
Distributions and correlations among nine direct and derived germination traits. Germination was defined as root emergence from seed. We directly measured Germination Rate (GR) on the 21st day under chilling conditions (GR21_C) and on the 7th day under normal conditions (GR_N). We also directly measured Days To 50% germination under Normal conditions (DT50_N) and Chilling conditions (DT50_C). We derived relative trait values by dividing chilling condition values by their corresponding normal condition values. In total, we evaluated nine germination traits, defined specifically as follows: GR21_C represents germination rate under chilling conditions (8 °C), measured at 21 days; GR_N (control) represents germination rate under normal conditions (25 °C), measured at 7 days; RGR represents relative rate of germination (GR21_C/GR_N); DT50_C represents days to 50% germination (up to 31 days) under chilling conditions; DT50_N (control) represents days to 50% germination under normal conditions; RDT50 represents relative days to 50% germination (DT50_C/DT50_N); GI_C represents germination index (GI = ∑(Gt/Tt), where Gt was the number of seeds newly germinated on day t and Tt was the number of days elapsed) under chilling conditions; GI_N represents germination index under normal conditions; and RGI represents relative germination index (GI_C/GI_N). Frequency distributions for each trait/index are illustrated as histograms in the center diagonal. Scatter plots of correlations and the numerical correlation coefficients between every two traits are shown in the areas below and above the diagonal, respectively. The red line in the scatter plots represents the correlation trend with diamonds displaying the medians.

In total, we measured nine traits, including the six combinations of three traits (GR, DT50, and GI) and two treatments (chilling and normal) plus the three relative traits derived from GR, DT50, and GI values. Seven of the nine traits had mono-modal distributions; both DT50_C and DT50_N displayed weak indications of bi-modal distributions (Fig. 2). Nevertheless, a robust statistical analysis was achieved by treating these traits as normally distributed and using a mixed linear model to further derive BLUPs. The overall inbred line performances relative to these nine traits were calculated as BLUPs, using the same method previously used to calculate the overall performances of 5,000 maize inbred lines while eliminating environment effects^26^.

Correlations were weak between each trait under chilling and normal conditions (Supplementary Table 2 and Fig. 2). The weak correlations suggest that germination under chilling conditions might be controlled by different genes. The three traits under chilling conditions were more highly correlated than under normal conditions. The two primary traits (RGR and RDT50) were characterized for their distributions within subpopulations defined in previous study^27^.

### Associated SNPs

**Figure 3:**
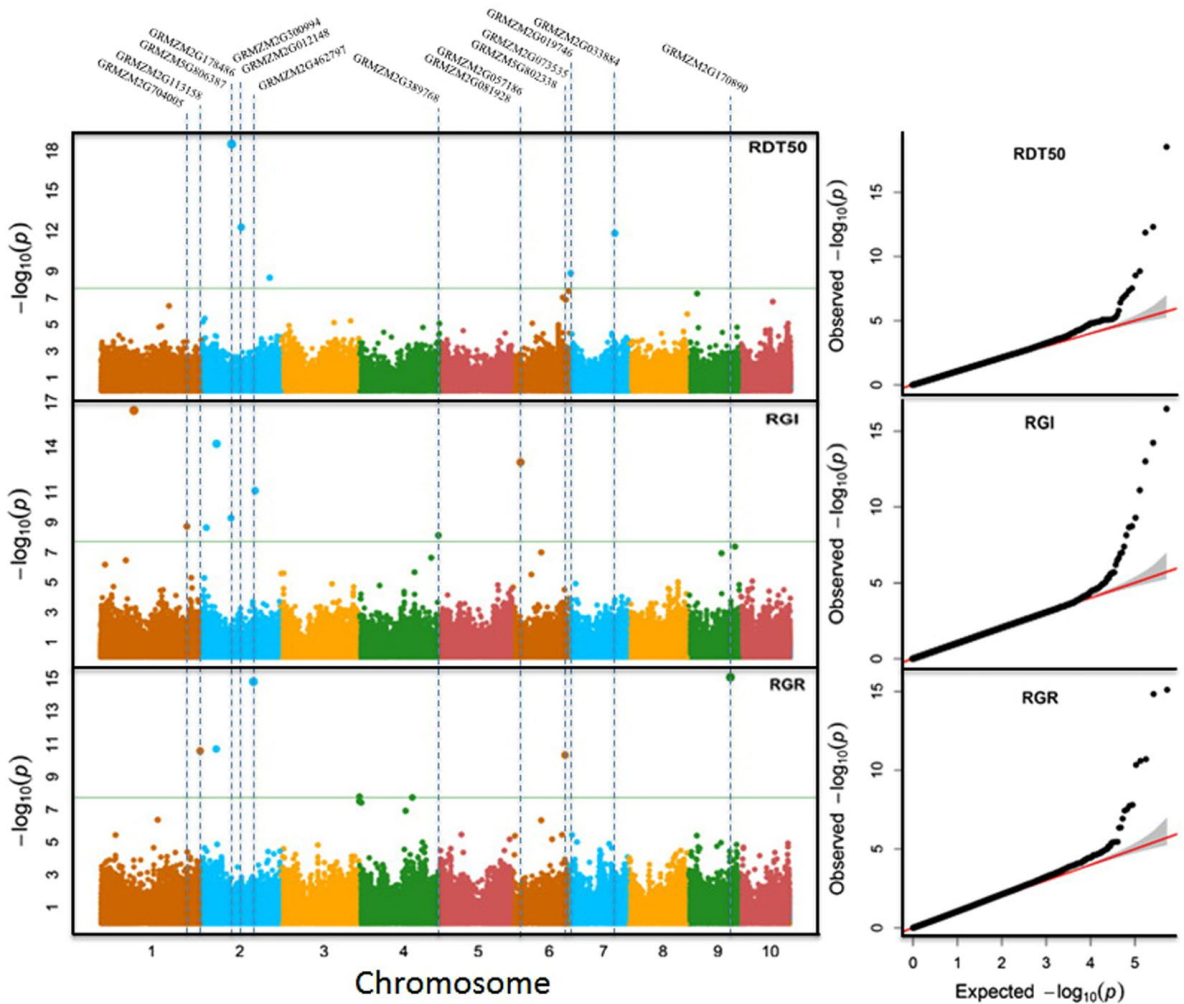
Manhattan and Quantile-Quantile plots of GWAS on three derived germination traits. The three traits (RDT50, RGI, and RGR) are the relative values derived from the germination trait values (DT50, GI, and GR) under chilling conditions divided by their corresponding trait values under normal conditions. Germination was defined as root emergence from seed. GWAS were performed on 241 inbred lines, genotyped with 2,271,584 SNPs, using the recently developed FarmCPU method and software package (http://zzlab.net/FarmCPU). The left panel displays the signals of associations across maize genome. The right panel demonstrates the overlapped and exceeded associations between the observed signals (black dots) and the expected (red lines) signals under the null hypotheses. GWAS identified a total of 17 SNPs that surpassed the Bonferroni threshold (horizontal green lines) for the three traits. One associated SNP was shared by RDT50 and RGI. Among the 17 associated SNPs, 11 were underlain by candidate genes (vertical dash lines) that were previously reported to be associated with cold tolerance.

GWAS were conducted on the BLUPs of the three relative traits (RGR, RDT50, and RGI) for 241 maize inbred lines genotyped by 2,271,584 SNPs, using recently developed FarmCPU method^28^ and software package (http://zzlab.net/FarmCPU). As the measurements were strongly associated with population structure (Supplementary Figure 2), the principal components derived from all the SNPs were fitted as covariates to control population structure in the association study. The P values were fully controlled without inflation, except very small proportions of SNPs that exceeded the null hypothesis expectation (2.2E-08) at a type I error of 5%. We identified 17 associated SNPs (Fig. 3 and Table 2) that surpassed the Bonferroni threshold. All SNPs had a Minor Allele Frequency (MAF) of 10% or above. The strongest associated SNP had a P value of 2.96E-19.

**Figure 4:**
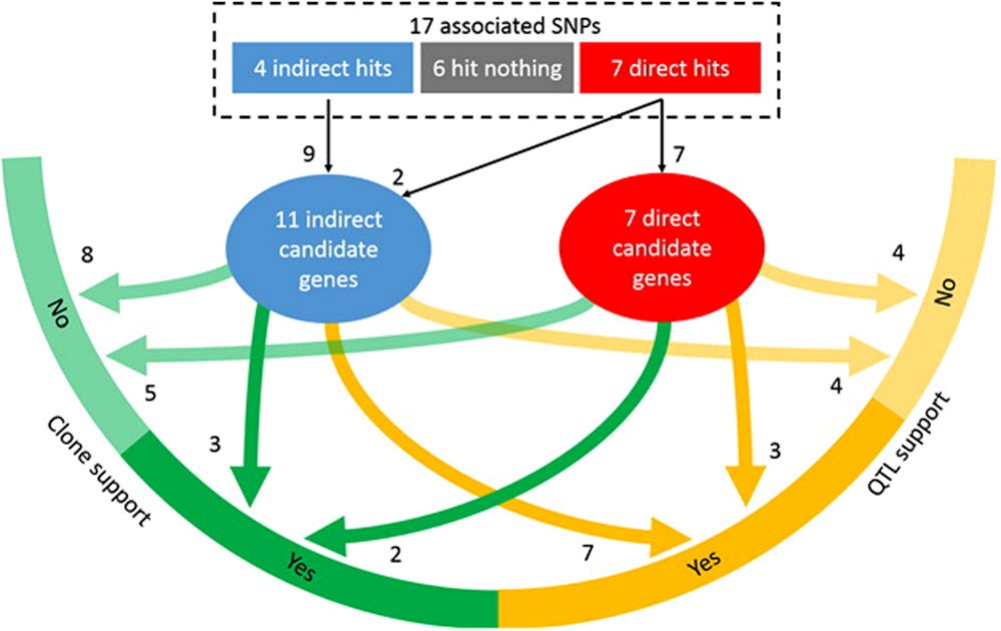
Diagram of associated SNPs, candidate genes, and support by QTL and gene cloning studies. In total, 18 candidate genes were identified for the 17 associated SNPs. Among these candidate genes, 10 were supported by QTL studies and five by gene cloning studies. Three genes were supported by both types of studies.

**Table 2:** Location, frequency and P values of associated SNPs.

SNP	Chromosome	Position	MAF	P value		
				RDT50	RGI	RGR
S1_258878734	1	258,878,734	0.42		1.84E-09	
S1_296660959	1	296,660,959	0.45			2.53E-11
ss196425965	1	101,270,646	0.49		3.44E-17	
S2_15690029	2	15,690,029	0.30		2.22E-09	
S2_43176376	2	43,176,376	0.10			1.98E-11
S2_46211425	2	46,211,425	0.39		5.82E-15	
S2_117871531	2	117,871,531	0.19	5.07E-13		
S2_154533439	2	154,533,439	0.13			1.45E-15
S2_161121602	2	161,121,602	0.08		7.66E-12	
S2_202342038	2	202,342,038	0.35	2.99E-09		
ss196436428	2	88,979,688	0.47	2.96E-19	5.07E-10	
S4_238122472	4	238,122,472	0.10		7.33E-09	
S6_156520680	6	156,520,680	0.50			4.59E-11
S6_23724609	6	23,724,609	0.28		9.70E-14	
S7_1956860	7	1,956,860	0.41	1.37E-09		
S7_134104928	7	134,104,928	0.15	1.38E-12		
S9_128655946	9	128,655,946	0.35			7.86E-16

The location is indicated by chromosome and base pair position. The frequency is indicated by the Minor Allele Frequency (MAF). The P values less than Bonferroni threshold (2.2E-8) corresponding to 5% type I error are displayed as scientific notations.

MAF: Minor Allele Frequency.

RGR: Relative germination rate (GR21_C/GR_N).

RDT50: Relative days to 50% root germination (DT50_C/DT50_N).

RGI: Relative germination index (GI_C/GI_N).

Among the 17 associated SNPs, only one SNP (ss196436428) was associated with two traits (RDT50 and RGI). Five SNPs were associated with RDT50; two SNPs were located on chromosome 7 and three were distributed on chromosome 2. Eight SNPs were associated with RGI and distributed on chromosomes 1, 2, 4, and 6. The remaining five SNPs were associated with RGR, with two located on chromosome 2, and one each distributed on chromosomes 1, 6, and 9.

### Comparison of associated SNPs and QTLs of cold-related traits

Among the 17 associated SNPs, five were located directly within QTL regions identified in previous studies and reported to be responsible for cold traits in maize (Supplementary Table 3). Two SNPs associated with RDT50 on chromosome 2 (S2_117871531 at 117,871,531 bp and ss196436428 at 88,979,688 bp) were located in QTL regions associated with ascorbate, Chlorophyll b, and Chlorophyll a + b content under suboptimal growing temperatures^14^; trapping efficiency of PSII (F′_v_/F′_m_), carbon exchange rate (CER) at 15 °C, and leaf greenness (SPAD) across temperatures^17^; and Fv/Fm across different sowing stages in a field experiment^29^.

One SNP associated with RGI and two SNPs associated with RGR corresponded to QTLs on chromosomes 1, 2, and 6. SNP S1_258878734 on chromosome 1 at 258,878,734 bp was detected in QTL regions associated with malic enzyme content at suboptimal temperature^14^, specific leaf area (SLA) at suboptimal temperature^12^, operating quantum efficiency of PSII photochemistry (ɸPSII) at 15 °C, and minimal fluorescence (Fo) across temperatures^17^. SNP S2_154533439 (chr2: 154,533,439 bp) was detected in a QTL region associated with CO_2_ fixation and ɸPSII in the third-leaf stage at 15 °C^11^. SNP S6_156520680 (chr6: 156,520,680 bp) was located in a QTL region associated with shoot nitrogen content (N%) under 15 °C growing conditions^17^. These findings suggested most of the detected SNPs (10 out of 18) are located within previously identified QTL regions associated with maize cold-related traits.

### Identification of candidate genes in maize

We used the B73 RefGen_v2 Maize Gene Database^30^ (http://www.maizegdb.org/) to identify candidate genes hit directly by associated SNPs or nearby genes in high LD with the associated SNPs. Among the 17 associated SNPs, seven had hits directly on candidate genes. An additional 11 candidate genes were identified with extended screening on genes in high LD (r^2^ ≥ 0.8) and within distance of 200 kb of four associated SNPs. The candidate genes of direct and indirect hits are summarized with table and figure (Table 3 and Fig. 4). Of the total 18 candidate genes identified, three contained the SNP associated with both RDT50 and RGI, six contained the SNPs associated with RDT50, five contained the SNPs associated with RGR, and four contained the SNPs associated with RGI. We were unable to find candidate genes that associated with six SNPs, including one SNP on chromosome 1 (ss196425965) and five SNPs on chromosome 2 (S2_15690029, S2_46211425, S2_161121602, S2_202342038, and S2_43176376). There is potential for new discovery of candidate genes around these SNPs with extended knowledge on gene functions.

**Table 3:** Functions of candidate genes associated with three derived germination traits.

Trait	SNP	Gene^*#^	Location	Distance (kb)	LD	Gene function
RGI	S1_258878734	GRMZM2G704005^#^	exon	0	1	Lactoylglutathione lyase
RGR	S1_296660959	GRMZM2G113158	exon	0	1	G-type lectin s-receptor-like serine threonine-protein kinase at1g34300
RDT50	S2_117871531	GRMZM2G318156^#^	exon	1.08	0.82	Uncharacterized protein LOC100502242
RDT50	S2_117871531	GRMZM2G012148^*#^	exon	47.62	0.8	Non-specific lipid-transfer protein at2g13820
RDT50	S2_117871531	GRMZM2G300994^#^	exon	−98.86	0.88	Gnat transcription partial
RDT50	S2_117871531	GRMZM5G871707^#^	exon	1.08	0.82	Uncharacterized protein LOC100502242
RGR	S2_154533439	GRMZM2G462797^#^	intron	0	1	TPA: hypothetical protein ZEAMMB73_597353
RGI	S4_238122472	GRMZM2G389768^*^	exon	0	1	Glycine-rich protein 2
RGR	S6_156520680	GRMZM2G073535^#^	exon	0	1	Protein translation factor sui1
RGR	S6_156520680	GRMZM5G802338^#^	exon	0.03	1	protein translation factor sui1
RGI	S6_23724609	GRMZM2G057186^*^	exon	0	1	Gdp-l-galactose phosphorylase 2-like
RGI	S6_23724609	GRMZM2G081928	exon	−199.88	0.84	Peroxidase
RDT50	S7_134104928	GRMZM2G033884	exon	−0.07	1	Pentatricopeptide repeat-containing protein mitochondrial
RDT50	S7_1956860	GRMZM2G019746	exon	0	1	AMP-dependent synthetase and ligase
RGR	S9_128655946	GRMZM2G170890	exon	−0.2	0.88	Mitochondrial fission 1 protein a-like
RDT50/RGI	ss196436428	GRMZM2G178486^*#^	exon	248.98	0.81	Zinc finger proteins: C3HC4-type family proteins
RDT50/RGI	ss196436428	GRMZM5G806387^*#^	exon	−366.49	0.8	Casp-like protein 2a1
RDT50/RGI	ss196436428	GRMZM2G148793^#^	intron	−216.19	0.86	TPA: hypothetical protein ZEAMMB73_617749

All candidate genes contain either an associated SNP or a SNP in high Linkage Disequilibrium (LD) (R Square ≥ 80%) with an associated SNP and within a distance <400 kb.

^*^ and ^#^ indicate the gene with supports of cloned genes and previous QTL studies, respectively.

### Pleiotropic SNPs

All the three traits investigated in this study are correlated with phenotypic correlation coefficient of 70% or above (Supplementary Table 2). Among them, RGR and GRI are more correlated (phenotypic correlation coefficient of 90%). Pleiotropic SNPs were observed among all the three traits, especially between RGR and GRI. The pleiotropic effect was demonstrated at both the levels of the SNPs with and without candidate genes identified.

The SNP tagged gene GRMZM2G178486 was above the Bonferroni significant threshold for both RDT50 and RGI (Fig. 3). The SNP tagged gene GRMZM2G462797 was above the threshold for both RGI and RGR. The SNP tagged gene GRMZM2G389768 was above the threshold for RGI and was the most significant SNP on chromosome 4 for RDT50 (8.52E-6). The SNP tagged gene GRMZM5G802338 was above the threshold for RGR and was the third significant SNP on chromosome 6 for both RDT50 (1.36E-7) and RGI (2.99E-5).

There were also SNPs that tagged multiple traits and these SNPs had been identified to be associate with candidate genes yet. The top significant SNP for RGI on chromosome 2 was the second significant SNP for RGR and the sixth SNP for RDT50. The top significant SNP for RGR on chromosome 5 was also the top significant for RDT50. The top significant SNP for RDT50 on chromosome 9 was the second significant SNP for RGR.

### Identification of candidate genes in analogous species

For each candidate gene identified in maize, we identified the homologous genes in *Arabidopsis* and rice. We also performed a comparative functional analysis of these corresponding genes (Table 4). Five of 18 candidate genes are predicted to respond to abiotic stress based on the known function of their Arabidopsis homologs. These five genes and the remaining 13 genes have been elucidated based on prediction (Supplementary Candidate Genes).

**Table 4:** Homologous genes in Arabidopsis and rice that correspond to the candidate genes associated with the three derived germination traits in maize.

No.	Gene ID	Gene name	Arabidopsis	Rice
1	GRMZM2G012148	ZEAMMB73_851804	AT3G22600.1	LOC_Os03g46150.2
2	GRMZM2G033884	ZEAMMB73_488923	AT5G19020.1	LOC_Os01g29430.1
3	GRMZM2G019746	ZEAMMB73_275546	AT5G63380.1	LOC_Os07g17970.1
4	GRMZM2G057186	ZEAMMB73_918708	AT5G55120.1,AT4G26850.1	LOC_Os12g08810
5	GRMZM2G073535	ZEAMMB73_586492	AT1G54290.1	LOC_Os05g41900.1
6	GRMZM2G081928	ZEAMMB73_289496	AT4G25980.1	LOC_Os12g08920.1
7	GRMZM2G170890	ZEAMMB73_122804	AT3G57090.1	LOC_Os03g24060.1
8	GRMZM2G178486	ZEAMMB73_014848	AT2G01150.1	LOC_Os04g16970.1
9	GRMZM2G300994	NA	AT2G32030.1	LOC_Os03g46200.1
10	GRMZM2G389768	csd2 - CSD-transcription factor 2	AT4G36020.1	LOC_Os02g02870.1
11	GRMZM2G462797	ZEAMMB73_597353	AT1G12650.1	LOC_Os09g35670.1
12	GRMZM2G704005	ZEAMMB73_876334	AT2G32090.1	LOC_Os03g45720
13	GRMZM5G802338	NA	NA	NA
14	GRMZM5G806387	ZEAMMB73_117651	AT1G17200.1	LOC_Os04g21320
15	GRMZM2G113158	ZEAMMB73_572941	AT1G34300.1	LOC_Os03g62180
16	GRMZM2G318156	ZEAMMB73_082780	NA	NA
17	GRMZM5G871707	NA	NA	NA
18	GRMZM2G148793	NA	NA	NA

### Functional prediction of candidate genes

We found 343 GO terms associated with the 18 candidate genes associated with the three traits (Supplementary Table 4). These GO terms belong to multiple functions. The first type of function is related to biological processes, including protein phosphorylation, metabolism, methylation, mitochondrial fission, proteolysis, and response to stimulus, brassinosteroid, oxidative stress, and transcription regulation. The second type is related to molecular functions, which involve lyase calmodulin, metal ion binding, zinc ion binding, adenyl-nucleotide exchange factor, and enzyme (N-acetyltransferase, GDP-D-glucose phosphorylase, peroxidase, methyltransferase, peptidase) and translation initiation factor activity. The third type of function is related to cellular components involving the plasma membrane and its integral components, mitochondrial matrix, plasma membrane, and extracellular regions.

We identified three candidate genes (GRMZM2G318156, GRMZM5G871707, and GRMZM2G148793) with unknown encoding functions; thus, gene ontology annotations were unavailable. From those genes with known functions, we observed that some candidate genes can be involved in multiple functions. For example, we found four genes (GRMZM2G462797, GRMZM2G081928, GRMZM2G389768, GRMZM2G113158) that are involved in all three functional types. Twelve genes are involved in two types, molecular function and biological process (Supplementary Table 4).

Most of the GO terms were specific to each trait (Supplementary Figure 3). Only 55.6%, 29.9%, and 27.2% were shared with other traits for RDT50, RGI, and RGR, respectively. Although RGR did not share any associated SNPs with the other two relative traits, we found 13 GO terms shared by all three traits. This finding suggests that these 13 GO terms have a greater chance for a stronger association with cold tolerance. Therefore, we detailed the relationships between the 18 candidate genes and these 13 GO terms (Supplementary Table 5 and Supplementary Figure 4).

## Discussion

Increasing maize yields in temperate zones by improving cold tolerance during germination and early growth stages is a complex process. This study focused on germination under chilling conditions; specifically, germination was defined as root emergence from seed. Experimental temperatures were also narrowly set to 25 °C for normal conditions and 8 °C for chilling conditions. Nevertheless, the 17 associated SNPs and 18 candidate genes we identified in this study might lead the direction for future studies. We found that 10 candidate genes were supported by QTL studies on traits related to cold tolerance, even though these previous studies measured their traits differently and at different growth stages. The overlap of five of our candidate genes with cloned genes for cold tolerance in maize, rice, and Arabidopsis also provided hints that genes related to cold tolerance may be shared across species^31–35^. This study provides valuable resources for future studies to enhance the understanding of the genetic architecture of maize for cold tolerance, and eventually, to improve maize varieties through breeding.

### Development of radicles under chilling condition

Root emergence under chilling conditions is the most important indicator of germination activation for cold tolerance^36^. We defined our chilling condition as 8 °C. Visible radicles were used as indicators of germination. Under the chilling condition, the 282-maize diverse panel exhibited a huge variation in the development of radicles, especially radicle length (Fig. 1). But, we only recorded germination as a binary trait (germinated or not) and the variation in length was not reported here. We observed a seemingly strong correlation between radicle length and germination rate. Even though, the genetic loci might be different from the length and could be controlled by different mechanisms of biological development.

### Germination analysis

Measuring GR at the optimal temperature, 25 °C, is a standard practice. Root emergence usually peaks at 2~3 days and germination is completed within 7 days^37^. Therefore, days/hours to reach 50% germination, DT50, and the total GR on the 7th day are the two typical measurements in germination studies. However, the dynamic process of germination is completely different under chilling conditions. For example, under the chilling condition (8 °C), not one line in the maize 282-diverse-panel exhibited 50% germination within 7 days.

Historically, GR on the 21st day has been used as the standard measurement under chilling conditions (8 °C), which corresponds to GR on the 7th day under normal conditions^38^. However, only about one-half of the lines in the maize diversity panel experienced a GR over 50% before the 21st day. For the majority of the remaining lines, germination continued for almost 31 days.

Using the GR at 21 days has two advantages. One advantage is that the methodology and analyses will be consistent with the existing literature. The other advantage is that the length of the experiment is reduced from 31 days to 21 days when GR on the 21st day is of interest. However, because a large portion of our lines were slow to germinate under chilling conditions, we extended our observations and used two complementary metrics—GR on the 21st day and number of days to reach 50% germination. The Pearson correlation coefficient was 85% in the maize 282-diverse-panel. We identified one SNP that was associated with both metrics. And, as expected, we also identified associated SNPs specific to each metric.

### Pleiotropy among three germination traits

We found strong correlations among the three relative traits derived from the three raw germination traits. The absolute values of Pearson correlations coefficients were all above 70% and 80% for phenotypic and genetic correlations, respectively. The absolute values of Pearson correlations coefficients between RGI and RGR were 90% and 97% for phenotypic and genetic correlations, respectively. More pleiotropic SNPs were found for the two traits (RGI and RGR) that are closely correlated.

### Priority of implications

This study did not include experimental data to evaluate candidate gene association with maize yield. We did not validate the expression and biological function for candidate genes either. Therefore, implications for future studies can only be prioritized by the confidence of our study results. We investigated multiple dimensions of confidence, including distance, pleiotropy, signal strength, linkage disequilibrium, cloning support, QTL analyses, and genes with GO terms shared with other candidate genes.

The null results include the six associated SNPs without candidate genes identified, eight candidate genes without support of QTLs, and three genes without GO terms identified. The possible causes include false positives, lack of recognition, and incomplete or incorrect gene annotation. These SNPs and genes have the lowest priority.

The Highest priority goes to five of the candidate genes (GRMZM2G389768, GRMZM2G057186, GRMZM2G012148, GRMZM2G178486, and GRMZM5G806387) that involve functions related to cold resistance, such as freezing tolerance response, ascorbate biosynthesis, and ABA, in maize or analogous species. Two of these five genes (GRMZM2G389768 and GRMZM2G057186) were hit directly, in the exonic region, by the associated SNPs identified in this study. The other three candidate genes (GRMZM2G178486, GRMZM5G806387, and GRMZM2G12148) were indirectly hit by two SNPs highly correlated and nearby associated SNPs (ss196436428 and s2_117871531) and were supported by previous QTL analyses (Fig. 4). The overlap among different studies suggested their potential values for future gene cloning studies and breeding to develop cold-tolerant maize varieties.

## Materials and Methods

### Plant materials and germination experiment

The plant seed materials originated from the USDA-ARS North Central Regional Plant Introduction Station, located in Ames, Iowa. The station collected maize diversity lines and made them accessible for public use. The core collection used in this study consisted of 282 maize lines and is commonly known as the “282 association panel”. This panel covers over 75% of the total variation for the entire maize collection, including tropical, subtropical, and temperate germplasms. In 2010, the 282 association panel was introduced into the Chinese Germplasm Bank, located at the Chinese Academy of Agricultural Sciences (CAAS) in Beijing, China. CAAS increased the size of this maize seed collection in 2011 and 2012.

To cover the environment requirements for seed production, we grew the CAAS-produced, 282 association panel seeds in both tropical and temperate regions to ensure seed quality in the extreme lines. Seeds were planted in China’s tropical Hainan province in winter 2013 and in temperate Heilongjiang province in spring 2014. Diseases, insects, and weeds were adequately controlled during the growing seasons. Physiologically mature ears were harvested from each environment, tropical and temperate. The seeds were dried under 25~30 °C conditions to achieve 14% water content and then stored at −4 °C. A total of 266 lines qualified for germination tests based on the quantity and physiological maturity status of seeds from both regions. Prior to seed germination experiments, all seeds were surface-disinfested with 1% NaOCl (sodium hypochlorite) for 5 minutes and then triple rinsed with sterile distilled water.

Following the ISTA protocol^37^, we conducted the experiments in two growth chambers (SANYO, MIR-253). The temperature was set at 8.0 °C^38^ for chilling conditions (treatment) and at 25.0 °C for normal conditions (control). Seeds were germinated in Petri dishes with sterile wetted filter paper. The Petri dishes were completely randomized. For each condition (treatment and control), we used three replicates per line and per batch of region where the seeds produced. Each replicate contained 30 seeds. Measurements were conducted daily, starting from day 1. We defined seed germination as observed root emergence. Following the advanced protocol of germination developed by Noli, E. *et al*.^39^ and Wen, W. *et al*.^38^, daily measurements spanned 7 days under normal conditions and 31 days under chilling conditions. The batches of seeds produced in two regions (temperate or tropical) were averaged for mapping genes underlying cold tolerance.

### Phenotypic data

We measured a total of nine phenotypic traits. The first six traits included the following: 1) germination (defined as root emergence) rate at 21 days under 8 °C (chilling) (GR21_C); 2) germination at 7 days under 25 °C (control) (GR_N); 3) relative germination rate (RGR), comparing chilling to control conditions (GR21_C/GR_N); 4) days to 50% germination under chilling (DT50_C); 5) days to 50% germination under control (DT50_N); and 6) relative days to 50% germination (RDT50), compared chilling to control conditions (DT50_C/DT50_N). The last three traits were based on a germination index (GI), calculated as GI = ∑(Gt/Tt), where Gt was the number of seeds newly germinated on day t and Tt was the number of days elapsed^40^. The GI under chilling conditions (GI_C) was calculated daily, from 0 to 31 days; the GI under control conditions (GI_N) was calculated daily, from 0 to 7 days (Mhatre and Chaphekar^41^). The relative GI (RGI) was used to compare chilling to control conditions (GI_C/GI_N).

For each trait above, the estimate breeding value (EBV) was calculated by the Best Linear Unbiased Prediction (BLUP) method. The genetic relationship matrix was estimated using about 51,742 SNPs. Together, the phenotypes of each trait and the genetic relationship matrix of individuals were used in rrBLUP to calculate each trait’s EBV^41–43^.

The standard deviation, range, mean, and median were calculated for each of the nine traits. EBV was used to assess genetic correlations between the traits. R language 3.12 and R package ‘psych’ was used to calculate correlations and to produce the correlation diagram^44^.

### Genotypic data

For genotypic data, we used a set of 51,742 SNP markers derived from an Illumina maize 50 K array^45^ and a GBS (genotyping by sequencing) dataset containing 2,219,845 SNP markers downloaded from http://mirrors.iplantcollaborative.org/browse/iplant/home/shared/panzea/genotypes/GBS/v23/Maize282_imputed_AllZea_GBS_Build_July_2012_FINAL.zip (accessed on September 12, 2016). The two datasets were combined and filtered for duplicate SNPs. A total of 2,271,584 SNPs, distributed over the entire maize genome, were used in this study.

### GWAS

Genome-wide association analysis on the three relative traits, RDT50, RGI, RGR, was performed using the FarmCPU method^28^. The first three principal components were fitted as the covariates to control population structure. The plots of the first three principal components reflected the subpopulation structure, including stiff stalk, non-stiff stalk and tropical/subtropical (Supplementary Figure 5). The threshold for detecting significant SNPs was determined by the Bonferroni multiple test correction at a type I error of 5%^46^.

### Identification of known QTLs

MaizeGDB was used to identify the QTLs on traits related to cold tolerance in maize. The intervals of the QTLs were defined by the starting and ending locations. The associated SNPs were compared with the intervals to examine the overlap between the linkage and association analyses.

### Identification of candidate genes

Genes hit directly by the associated SNPs and genes in high linkage disequilibrium (LD) with the associated SNPs and within 400 kb were selected as candidate genes. We used the PLINK analysis toolset (http://pngu.mgh.harvard.edu/~purcell/plink/) to calculate LD as the squared correlation coefficient (r^2^) between each marker within an intra-chromosomal distance of 1000 kb. A histogram was then plotted to analyze the LD decay according genetic distances and pairwise LD across the entire genome. SNPs with a paired r^2^ ≥ 0.8 were identified within a 400 kb region up- and downstream of the significant cold-trait-associated SNPs. Then, we examined all candidate genes either containing the associated SNPs or in high linkage disequilibrium (r^2^ ≥ 0.8) with the associated SNPs and within a distance of 400 kb. The B73 RefGen_V2 gene model from the maizeGDB website (http://www.maizegdb.org/) was used to map the loci and genetic information.

### Annotation of candidate genes

Based on the complementary DNA (cDNA) sequences of the candidate genes, we used previously published evidence and the National Center for Biotechnology Information (NCBI) database (http://www.ncbi.nlm.nih.gov/) to search for gene annotations and functions in related cereals and ontology plants. The gene ontology (GO) for candidate genes was performed by AgriGO (http://bioinfo.cau.edu.cn/agriGO/analysis.php)^47^ and Blast2GO 3.0 software^48^.

## Electronic supplementary material

Supplementary Candidate Genes, Tables, Figures
