# Supplementary material for: Genome-wide association study Identified multiple Genetic Loci on Chilling Resistance During Germination in Maize
Source: Sci Rep. 2017 Sep 7;7:10840. doi: 10.1038/s41598-017-11318-6 (PMC5589824; doi:10.1038/s41598-017-11318-6)
Supplement: Supplementary file 1 — Supplementary Candidate Genes, Tables, Figures [file 41598_2017_11318_MOESM1_ESM.doc]

**Genome-wide association study Identifies multiple Genetic Loci on Chilling Resistance during Germination in Maize**

Guanghui Hu^1,2,3#^, Zhao Li^1,3#^, Yuncai Lu^4^, Chunxia Li^2^, Shichen Gong^2^, Shuqin Yan^2^, Guoliang Li^2^, Mingquan Wang^2^, Honglei Ren^2^, Haitao Guan^5^, Zhengwei Zhang^4^, Dongling Qin^1^, Mengzhu Chai^1^，Juping Yu^1^，Yu Li^6^, Deguang Yang^1*^, Tianyu Wang^6*^, Zhiwu Zhang^1,3*^

*^1^* *College of Agriculture, Northeast Agricultural University, Harbin 150030, Heilongjiang, China.*

*^2^* *Institute of Maize Research, Heilongjiang Academy of Agricultural Sciences, Harbin 150086, Heilongjiang, China.*

*^3^* *Department of Crop and Soil Sciences, Washington State University,* *Pullman 99163, WA, USA.*

*^4^**College of Agricultural Resources and Environment, Heilongjiang University, Harbin 150001, Heilongjiang, China.*

*^5^* *Quality & Safety Inst. of Agricultural Products, Heilongjiang Academy of Agricultural Sciences, Harbin 150086, Heilongjiang, China.*

*^6^Institute of Crop Science, Chinese Academy of Agricultural Sciences，Beijing* *100081, China.*

^#^Contributed equally to this work.

^*^Corresponding author.

Deguang Yang; E-mail: [ydgl@tom.com](mailto:ydgl@tom.com) (DY)

Tianyu Wang; E-mail: [wangtianyu@263.net](mailto:wangtianyu@263.net ) (TW)

Zhiwu Zhang; E-mail: [Zhiwu.Zhang@wsu.edu](mailto:Zhiwu.Zhang@wsu.edu) (ZZ)

**Supplementary Candidate Genes**

**GRMZM2G389768 (*csd2*)**: The exonic region of candidate gene GRMZM2G389768 on chromosome 4 contains SNP S4_23812247 (significantly associated with RGI), encodes glycine-rich protein and shows homology to cold shock domain-containing proteins (CSD)(<http://www.maizegdb.org/>). In molecular biology, CSD is constituted by about 70 amino acids involved in function of prokaryotic and eukaryotic DNA-binding proteins^1,2^. The expression of GRMZM2G389768 is down-regulated under cold stress, but changes little under the stress of salt or abscisic acid (ABA). A recent study showed that GRMZM2G389768 is also highly expressed in different plant tissues, suggesting that CSDs are actively participating during the lifetime of maize ^3^. GRMZM2G389768 has also been found in strong correlation with multiple traits including blade size, shoot, character of timing in maize RIL population ^4,5^. GRMZM2G389768 best hits gene AT4G36020 in Arabidopsis. AT4G36020 and similar genes encode CSD proteins, such as Cold shock domain protein 1(CSDP1), Cold shock protein 1(CSP1), Arabidopsis thaliana CSP 1(ATCSP1) ([http://www.arabidopsis.org](http://www.arabidopsis.org/)). These proteins aid in cold acclimation by blocking mRNAs on energy-consuming process which can accelerate translation at cold temperatures (http://www.ncbi.nlm.nih.gov) ^6^.

In E.coli , the expression of *CSDP1* was markedly up-regulated by cold treatment, but transcript levels of *CSDP1* were down-regulated by dehydration stress and salt stress ^7^. The *CSDP1*-expressing plants had higher freezing tolerance than the mutant plants, based on survival rate under freezing stress. These results illuminate that *CSDP1* may suppress the cold sensitivity of *E.coli* cells, and may play a positive role in stimulating growth under cold treatment ^8^.

**GRMZM2G057186**: The exonic region of candidate gene GRMZM2G057186, also located on chromosome 6, contains significant SNP S6_23724609 (associated with RGI). Two Arabidopsis genes, vitamin C defective 2 (VTC2: At4g26850) and VTC2-homologue (VTC5: At5g55120), are best hits to this maize gene (http://www.maizegdb.org/). At4g26850 encodes a new protein that participates in ascorbate biosynthesis ([http://www.arabidopsis.org](http://www.arabidopsis.org/)). At5g55120 encodes a GDP-L-galactose phosphorylase, with similar biochemical properties as *VTC2* ([http://www.arabidopsis.org](http://www.arabidopsis.org/)), and plays a major role in ascorbate biosynthesis which can influence survival rate of Arabidopsis as ascorbic acid deficient mutants ^9,10^.

The expression of these two genes, At4g26850(*VTC2*) and At5g55120(*VTC5*), was increased by treatment with jasmonates, which induces the accumulation of ascorbate ^11^. Ascorbate bioysnthesis is an important response to oxidative stress, which affects the antioxidant defense system that influences maize seed germination at low temperatures ^12^. Other research suggested that *VTC5* and *VTC2* mediate ascrobate metabolic pathways, which can provide resistance when induced by abiotic stress or environmental stresses ^11^. Additionally, GRMZM2G057186 best hits rice gene LOC_Os12g08810 (OsVTC2), which can elevate transcripts of OsVTC2 in rice leaves under cold stress ^13^.

**GRMZM2G178486**: The exonic region of candidate gene GRMZM2G178486, also on chromosome 2, contains a highly correlated SNP nearby significant SNP ss196436428 (associated with RDT50 and RGI). This gene encodes zinc finger proteins and C3HC4-type family proteins in maize (http://ensembl.gramene.org/Zea_mays/Transcript). GRMZM2G178486 best hits Arabidopsis gene At2g01150, which encodes RING-H2 finger protein (RHA2B) and has expression in multiple plant tissues such as vascular, root tips, and so on (<http://www.arabidopsis.org/>). At2g01150 is involved in protein degradation through encoding RING-H2 finger proteins and shows strong shade responsiveness ^14^. The transcripts of Ring-H2 finger protein 2B/At2g01150 significantly increased after treatment of sucrose readdition ^15^. A study of carbon status in Arabidopsis proposed that the family protein of zinc finger (At2g01150) is responsible for the plant's resistant to abiotic stress ^16^. RHA2B ([At2g01150](http://www.plantphysiol.org/external-ref?link_type=GEN&access_num=At2g01150)), the closest homolog of RHA2A, plays a crucial role in ABA signaling. The expression of RHA2B is induced as ABA effect; overexpression results in variation on phenotype-associated ABA, such as hypersensitivity of ABA in early stages of germination and seedling development ^17^. Previous research found that ABA performs an important role in plant developmental stages, including seed germination, seedling growth, etc. ABA can also mediate an increase in plant adaptability to various abiotic stress, especially cold, drought, and salt ^17–20^.

Additionally, GRMZM2G178486 best hits rice gene LOC_Os04g16970, which is involved in several biological processes and molecular functions, including response to abiotic stimuli (http://rice.plantbiology.msu.edu).

**GRMZM5G806387**: The exonic region of candidate gene GRMZM5G806387, also on chromosome 2, contains another highly correlated SNP nearby significant SNP ss196436428 (associated with RDT50 and RGI). It’s protein family contains a domain that may be involve a catalytic activity (PFAM ID: [PF04535](http://pfam.xfam.org/family/PF04535)). This gene best hits Arabidopsis gene At1g7200.1, involved in response to karrikins, a group of plant growth regulators associated with seed germination ^21^. Expression of At1g7200.1 has an important role in chloroplast development. For example, if the gene expression is deficient, Arabidopsis leaves will exhibit a pale green color ^22^. This phenomenon also occurs in maize and other plants when subject to cold conditions. Additionally, GRMZM5G806387 best hits rice gene LOC_Os04g21320.1, which is generally involved in response to abiotic stimulus ([http://rice.plantbiology.msu.edu](http://rice.plantbiology.msu.edu/)).

**GRMZM2G012148**: The exonic region of candidate gene GRMZM2G012148, located on chromosome 2, contains a highly correlated SNP nearby significant SNP S2_117871531 (associated with RDT50). This gene encodes non-specific lipid-transfer proteins (nsLTPs) that participate in the functioning of biological processes, especially pathogen defense and abiotic stress response (http://www.maizegdb.org/). Previous research found that nsLTPs regulate the response under drought, salt, and cold threat conditions through transcript abundance in the maize nsLTP gene family ^23^. GRMZM2G012148 best hits Arabidopsis AT3G22600.1, which also encodes LTPs (<http://blast.ncbi.nlm.nih.gov/>). Sasaki found that gene AT3G22600.1 may induce freezing tolerance in lag phase cells for transgenetic plants after 2 days of cold treatment^24^.

**GRMZM2G704005**. The exonic region of candidate gene GRMZM2G704005, located on chromosome 1, contains significant SNP S1_258878734 (associated with RGI). This gene encodes the lactoylglutathione lyase/glyoxalase I family protein (PFRM ID: PF00903). GRMZM2G704005 best hits Arabodopsis AT2G32090.1, which can experience down-regulated gene expression under threat of salt and drought ^25^. GRMZM2G704005 best hits gene LOC.Os03g45720 in rice, which can experience up-regulated gene expression during redox homeostasis ^26^.

**GRMZM2G113158.** The exonic region of candidate gene GRMZM2G113158, also located on chromosome 1, contains the significant SNP S1_296660959 (associated with RGR). This gene encodes the lectin protein kinase family protein (PFRM ID: PF00069). GRMZM2G113158 best hits AT1G34300.1 on Arabidopsis, which is involved in protein amino acid phosphorylation and pollen recognition, and experiences up-regulated gene expression when treated with ABA and salt stress ^27^.

**GRMZM2G462797.** The intronic region of candidate gene GRMZM2G462797, located chromosome 2, contains significant SNP S2_ 154533439 (associated with RGR). The function of this gene is unknown in maize and in other species.

**GRMZM2G073535**. The exonic region of candidate gene GRMZM2G073535, located on chromosome 6, contains significant SNP S6_156520680 (associated with RGR). This gene encodes protein translation factor SUI1 (<http://www.maizegdb.org/)> (PFAM ID: PF01253). GRMZM2G073535 best hits Arabidopsis AT1G54290.1, which was found to inhibit pollen and seed germination when treated with the drug cycloheximide ^28^.

**GRMZM2G019746.** The exonic region of candidate gene GRMZM2G019746, located on chromosome 7, contains significant SNP S7_1956860 (associated with RDT50).This gene encodes AMP-dependent synthetase and ligase and is also involved in flavonoid biosynthesis in maize kernels ^29^ ( PFAM ID: PF00501).. GRMZM2G019746 best hits Arabidopsis AT5G63380.1, which is involved in multiple functions of annotation, such as encoding a peroxisomal protein that can activate fatty acids by esterification with Coenzyme A(CoA) ^30,31^. AT5G63380.1 is also involved in jasmonic acid biosynthesis (<http://www.arabidopsis.org/servlets/TairObject?type=gene&name=AT5G63380.1>)

**GRMZM2G148793.** The intronic region of candidate gene GRMZM2G148793, also on chromosome 2, contains a highly correlated SNP nearby significant SNP ss196436428 (associated with RDT50). The encoding function for this gene is also unknown. Two other genes, GRMZM2G178486 and GRMZM5G806387, are also in high LD with the same significant SNP, ss196436428, as GRMZM2G148793. But, all three genes are located much farther from ss196436428 compared to the distances between other candidate genes and their associated SNPs (Table 3).

**GRMZM2G300994.** The exonic region of candidate gene GRMZM2G300994, also located on chromosome 2, contains another highly correlated SNP nearby significant SNP S2_117871531 (associated with RDT50). This gene encodes gnat transcription partial, with N-acetyltransferase activity, and is involved in metabolic processes (http://www.maizegdb.org/)，and its ontology term show the only one function related to primary root (http://purl.obolibrary.org/obo/PO_0020127).

**GRMZM2G318156 and GRMZM5G871707.** The exonic regions of candidate genes GRMZM2G318156 and GRMZM5G871707, also located on chromosome 2, each contain a highly correlated SNP nearby significant SNP S2_11787153 (associated with RDT50). However, the encoding functions for these genes have yet to be characterized, and gene ontology annotations are unavailable. **GRMZM5G802338.** The exonic region of candidate gene GRMZM5G802338, located on chromosome 6, contains a highly correlated SNP nearby significant SNP S6_156520680 (associated with RGR). Similar to candidate gene GRMZM2G073535, this gene encodes protein translation factor SUI1 (http://www.maizegdb.org). We could not find previous research about the function or a homologous species for GRMZM5G802338.

**GRMZM2G081928.** The exonic region of candidate gene GRMZM2G081928, located on chromosome 6, contains a highly correlated SNP nearby significant SNP S6_23724609 (associated with RGI). This gene encodes peroxidase and involved in plant response to oxidative stress (http://www.maizegdb.org) ( PFAM ID: PF00141). GRMZM2G081928 best hits Arabidopsis gene AT4G25980.1, which is expressed in shoot apex, embryo, and seeds (<https://www.arabidopsis.org/>).

**GRMZM2G033884**. The exonic region of candidate gene GRMZM2G033884 on chromosome 7 contains a highly correlated SNP nearby significant SNP S7_134104928 (associated with RDT50). GRMZM2G033884 best hits Arabidopsis gene AT5G19020.1, which encodes a pentatricopeptide repeat-containing protein involved in mitochondrial mRNA editing (<https://www.arabidopsis.org/)> ( PFAM ID: PF01535)..

**GRMZM2G170890.** The exonic region of candidate gene GRMZM2G170890 on chromosome 9 contains a highly correlated SNP nearby significant SNP S9_128655946 (associated with RGR). This gene encodes mitochondria fission 1 (FIS) protein (http://www.maizegdb.org). GRMZM2G170890 best hits Arabidopsis gene AT3G57090.1, which also encodes a protein similar to yeast FIS proteins that are involved in peroxisome division (<https://www.arabidopsis.org/>).

**References**

1. Jones, P. G. & Inouye, M. The coJones, P. G., & Inouye, M. (1994). The cold-shock response--a hot topic. *Mol. Microbiol.* **11,** 811–818 (1994).

2. Doniger, J., Landsman, D., Gonda, M. A. & Wistow, G. The product of unr, the highly conserved gene upstream of N-ras, contains multiple repeats similar to the cold-shock domain (CSD), a putative DNA-binding motif. *New Biol* **4,** 389–395 (1992).

3. Zhang, J., Zhao, Y., Xiao, H., Zheng, Y. & Yue, B. Genome-wide identification, evolution, and expression analysis of RNA-binding glycine-rich protein family in maize. *J. Integr. Plant Biol.* **56,** 1020–31 (2014).

4. Baute, J. *et al.* Correlation analysis of the transcriptome of growing leaves with mature leaf parameters in a maize RIL population. *Genome Biol.* **16,** 168-193 (2015).

5. Baute, J. *et al.* Combined large-scale phenotyping and transcriptomics in maize reveals a robust growth regulatory network. *Plant Physiol.* **170,** 1848-1867 (2016).

6. Juntawong, P., Sorenson, R. & Bailey-Serres, J. Cold shock protein 1 chaperones mRNAs during translation in *Arabidopsis thaliana*. *Plant J.* **74,** 1016–1028 (2013).

7. Kim, J. S. *et al.* Cold shock domain proteins and glycine-rich RNA-binding proteins from *Arabidopsis thaliana* can promote the cold adaptation process in *Escherichia coli*. *Nucleic Acids Res.* **35,** 506–516 (2007).

8. Park, S. J., Kwak, K. J., Oh, T. R., Kim, Y. O. & Kang, H. Cold shock domain proteins affect seed germination and growth of *Arabidopsis thaliana* under abiotic stress conditions. *Plant Cell Physiol.* **50,** 869–878 (2009).

9. Majláth, I., Szalai, G. & Janda, T. Exploration of cold signalling related to ascorbate and salicylic acid in *Arabidopsis thaliana*. *Acta Biol. Szeged.* **55,** 117–118 (2011).

10. Dowdle, J., Ishikawa, T., Gatzek, S., Rolinski, S. & Smirnoff, N. Two genes in *Arabidopsis thaliana* encoding GDP-l-galactose phosphorylase are required for ascorbate biosynthesis and seedling viability. *Plant J.* **52,** 673–689 (2007).

11. Sasaki-Sekimoto, Y. *et al.* Coordinated activation of metabolic pathways for antioxidants and defence compounds by jasmonates and their roles in stress tolerance in Arabidopsis. *Plant J.* **44,** 653–668 (2005).

12. Ijaz Ahmad,. Effect of seed priming with ascorbic acid, salicylic acid and hydrogen peroxide on emergence, vigor and antioxidant activities of maize. *African J. Biotechnol.* **11,** (2012).

13. Jo, Y. & Hyun, T. K. Genome-wide identification of antioxidant component biosynthetic enzymes: Comprehensive analysis of ascorbic acid and tocochromanols biosynthetic genes in rice. *Comput. Biol. Chem.* **35,** 261–268 (2011).

14. Devlin, P., Yanovsky, M. & Kay, S. A genomic analysis of the shade avoidance response in Arabidopsis. *Plant Physiol.* **133,** 1617–1629 (2003).

15. Cookson, S. J. *et al.* Temporal kinetics of the transcriptional response to carbon depletion and sucrose readdition in Arabidopsis seedlings. *Plant. Cell Environ.* **39,**768–786 (2015).

16. Usadel, B. *et al.* Global Transcript Levels Respond to Small Changes of the Carbon Status during Progressive Exhaustion of Carbohydrates in Arabidopsis Rosettes. *Plant Physiol.* **146,** 1834–1861 (2008).

17. Li, H. *et al.* The Arabidopsis RING finger E3 ligase RHA2b acts additively with RHA2a in regulating abscisic acid signaling and drought response. *Plant Physiol.* **156,** 550–63 (2011).

18. Finkelstein, R. R., Gampala, S. S. L. & Rock, C. D. Abscisic acid signaling in seeds and seedlings. *Plant Cell* **14 Suppl,** S15–S45 (2002).

19. Koornneef, M., Hanhart, C. J., Hilhorst, H. W. & Karssen, C. M. In Vivo Inhibition of Seed Development and Reserve Protein Accumulation in Recombinants of Abscisic Acid Biosynthesis and Responsiveness Mutants in *Arabidopsis thaliana*. *Plant Physiol.* **90,** 463–469 (1989).

20. Leung, J. & Giraudat, J. Abscisic Acid Signal Transduction. *Annu. Rev. Plant Physiol. Plant Mol. Biol.* **49,** 199–222 (1998).

21. Chiwocha, S. D. S. *et al.* Karrikins: A new family of plant growth regulators in smoke. *Plant Sci.* **177,** 252–256 (2009).

22. Olinares, P. D. B., Ponnala, L. & van Wijk, K. J. Megadalton Complexes in the Chloroplast Stroma of *Arabidopsis thaliana* Characterized by Size Exclusion Chromatography, Mass Spectrometry, and Hierarchical Clustering. *Mol. Cell. Proteomics* **9,** 1594–1615 (2010).

23. Wei, K. & Zhong, X. Non-specific lipid transfer proteins in maize. *BMC plant biology* **14,** 281 (2014).

24. Sasaki, Y. *et al.* Characterization of growth-phase-specific responses to cold in *Arabidopsis thaliana* suspension-cultured cells. *Plant Cell Environ.* **31,** 354–365 (2008).

25. Cho, S.-M., Kang, B. R. & Kim, Y. C. Transcriptome Analysis of Induced Systemic Drought Tolerance Elicited by Pseudomonas chlororaphis O6 in *Arabidopsis thaliana*. *plant Pathol. J.* **29,** 209–20 (2013).

26. Han, C., He, D., Li, M. & Yang, P. In-depth proteomic analysis of rice embryo reveals its important roles in seed germination. *Igarss 2014* **5,** 1–5 (2014).

27. Cl, I.- & Gepstein, S. *( 19 ) United States*. **1,** (2013).

28. Salmi, M., Bushart, T., Stout, S. & Roux, S. Profile and analysis of gene expression changes during early development in germinating spores of Ceratopteris richardii. *Plant Physiol.* **138,** 1734–1745 (2005).

29. Wen, W. *et al.* Metabolome-based genome-wide association study of maize kernel leads to novel biochemical insights. *Nat. Commun.* **5,** 3438 (2014).

30. Suzuki, S. *et al.* Analysis of expressed sequence tags in developing secondary xylem and shoot of Acacia mangium. *J. Wood Sci.* **57,** 40–46 (2011).

31. Yi, S. Y. *et al.* Microarray Analysis of bacterial blight resistance 1 mutant rice infected with Xanthomonas oryzae pv. oryzae. *Plant Breed. Biotechnol.* **1,** 354–365 (2013).

**Supplementary Table**

**Supplementary Table 1.** Parameter estimates of the Boltzmann fitting curve on days to 50% germination (DT50) under chilling conditions. The Boltzmann function includes four parameters: y = A_2_ + (A_1_-A_2_)/(1 + exp((x-x_0_)/dx)), where y is the observed DT50; A_1_ and A_2_ are the initial and final DT50 values, respectively; and x is the cumulative frequency corresponding to y.

| **Parameters** | **Description** | **Estimates** | **Standard Error** |
| --- | --- | --- | --- |
| A_1_ | Initial value | 10.32237 | 0.25098 |
| A_2_ | Final value | 29.63601 | 0.20278 |
| x_0_ | Center | 0.48641 | 0.00421 |
| dx | Slope | 0.11815 | 0.00436 |

**Supplementary Table 2.** Phenotypic and genetic correlations among nine direct and derived germination traits. The phenotypic correlations were calculated on the average phenotype value across replicates (lower triangle). The genetic correlations were calculated on the Best Linear Unbiased Prediction (BLUP) of inbred lines across replicates (upper triangle).

| Traits | GR21_C | GR_N | RGR | DT50_C | DT50_N | RDT50 | GI_C | GI_N | RGI |
| --- | --- | --- | --- | --- | --- | --- | --- | --- | --- |
| GR21_C | 1 | 0.42^**^ | 0.97^**^ | -0.99^**^ | -0.16 | -0.85^**^ | 0.98^**^ | 0.31^**^ | 0.97^**^ |
| GR_N | 0.23^**^ | 1 | 0.31^**^ | -0.45^**^ | -0.58^**^ | -0.08 | 0.44^**^ | 0.86** | 0.33^**^ |
| RGR | 0.87^**^ | -0.09 | 1 | -0.95^**^ | 0.11 | -0.86^**^ | 0.94^**^ | 0.20 | 0.97^**^ |
| DT50_C | -0.98^**^ | -0.26^**^ | -0.83^**^ | 1 | 0.19 | 0.85^**^ | -0.97^**^ | -0.34^**^ | -0.96^**^ |
| DT50_N | -0.12 | -0.6^**^ | 0.2^**^ | 0.16^**^ | 1 | -0.27^**^ | -0.15 | -0.64^**^ | 0.07 |
| RDT50 | -0.77^**^ | 0.07 | -0.75^**^ | 0.77^**^ | -0.38^**^ | 1 | -0.84^**^ | 0.05 | -0.87^**^ |
| GI_C | 0.97^**^ | 0.24^**^ | 0.82^**^ | -0.97^**^ | -0.15 | -0.75^**^ | 1 | 0.32^**^ | 0.97^**^ |
| GI_N | 0.21^**^ | 0.82^**^ | -0.08 | -0.24^**^ | -0.62^**^ | 0.09 | 0.23^**^ | 1 | 0.19 |
| RGI | 0.89^**^ | 0.04 | 0.9^**^ | -0.87^**^ | 0.04 | -0.73^**^ | 0.91^**^ | -0.03 | 1 |

GR21_C = Germination (root emergence) rate at 21 days under 8°C (chilling conditions).

GR_N = Germination rate at 7 days under normal conditions, 25°C (control).

RGR = Relative germination rate, comparing chilling to normal conditions (GR21_C/GR_N).

DT50_C = Days to 50% germination under chilling conditions.

DT50_N = Days to 50% germination under normal (control) conditions.

RDT50 = Relative days to 50% germination, comparing chilling to normal conditions (DT50_C/DT50_N).

GI_C = Germination index from 0 to 31 days under chilling conditions.

GI_N = Germination index from 0 to 7 days under normal (control) conditions.

RGI = Relative germination index, comparing chilling to normal conditions.

**Supplementary Table 3 The comparison for results of GWAS and QTL previous study on maize cold tolerance**

| **Significant SNP by GWAS** | | | **Result by QTL mapping** | | | | | | | |
| --- | --- | --- | --- | --- | --- | --- | --- | --- | --- | --- |
| **SNP ID** | **Chromsome and position** | **Traits** | **Bin** | **Nearest marker** | **QTL interval** | **Traits** | **Threaten conditions** | **Parental lines** | **Population size** | **Mapping method** |
| S6_156520680 | chr6: 156,520,680 | RGR | BIN6.06 | umc1859 | 153,956,114~161,325,348 | N(%) | 15°C | ETH-DH7;ETH-DL3 | F_2:3_:226 | CIM* |
| S2_117871531  /ss196436428 | chr2: 117,871,531  /chr2:88,979,688 | RDT50  /RDT50,RGI | BIN 2.05 | dupssr21 | 71,742,767~153,140,073 | Antioxidants | suboptimal temperature | ETH-DH7;ETH-DL3 | F_2:3_:226 | CIM |
|  |  |  |  | bnlg1909 |  | Chl b | suboptimal temperature | ETH-DH7;ETH-DL3 | F_2:3_:226 | CIM |
|  |  |  |  | bnlg1909 |  | Chl a+b | suboptimal temperature | ETH-DH7;ETH-DL3 | F_2:3_:226 | CIM |
|  |  |  |  | bnlg1909 |  | F^0^_v_/F^0^_m_ | 15°C | ETH-DH7;ETH-DL3 | F_2:3_:226 | CIM |
|  |  |  |  | bnlg1909 |  | CER | 15°C | ETH-DH7;ETH-DL3 | F_2:3_:226 | CIM |
|  |  |  |  | bnlg1909 |  | SPAD | 15°C | ETH-DH7;ETH-DL3 | F_2:3_:226 | CIM |
|  |  |  |  | bnlg1909 |  | SPAD | across temperature | ETH-DH7;ETH-DL3 | F_2:3_:226 | CIM |
|  |  |  |  | dupssr21 |  | Fv/Fm | different sowing stage | ETH-DH7;ETH-DL3 | F_2:3_:226 | CIM |
| S1_258878734 | chr1: 258,878,734 | RGI | BIN 1.09 | *bnlg1502* | 250,153,976~267,926,978 | ME | suboptimal temperature | ETH-DH7;ETH-DL3 | F_2:3_:226 | CIM |
|  |  |  |  | bnlg1502 |  | SLA | 15/13°C | ETH-DH7;ETH-DL3 | F_2:3_:226 | CIM |
|  |  |  |  | bnlg1502 |  | ɸpsii | 15°C | ETH-DH7;ETH-DL3 | F_2:3_:226 | CIM |
|  |  |  |  | bnlg1886 |  | F_0_ | across temperatures | ETH-DH7;ETH-DL3 | F_2:3_:226 | CIM |
| S2_154533439 | chr2:154533439 | RGR | BIN2.06 | umc98 | 153,140,073~187,179,001 | CO_2_ fixation | 15 °C | AC7643;AC7729/TZSRW | RILs:233 | CIM |
|  |  |  |  | umc98 |  | ɸPSII | 15 °C | AC7643;AC7729/TZSRW | RILs:233 | CIM |

^*^Composite interval mapping (CIM)

**Supplementary Table 4.** Gene ontology terms characterizing the candidate genes associated with three derived germination traits.

| **Gene ID** | **Gene ontology function** |
| --- | --- |
| GRMZM2G704005 | Lyase activity, phosphatidylinositol biosynthetic process, response to brassinosteroid |
| GRMZM2G113158 | Membrane, protein serine/threonine kinase activity, calmodulin binding, ATP binding, protein phosphorylation, protein N-linked glycosylation, recognition of pollen |
| GRMZM2G462797 | Mitochondrial matrix, adenyl-nucleotide exchange factor activity, regulation of catalytic activity, response to stimulus |
| GRMZM2G300994 | N-acetyltransferase activity, metabolic process |
| GRMZM2G318156 | Unknown |
| GRMZM5G871707 | Unknown |
| GRMZM5G806387 | Plasma membrane, integral component of membrane |
| GRMZM2G148793 | Unknown |
| GRMZM2G178486 | Zinc ion binding |
| GRMZM2G389768 | Nucleus, cytoplasm, proteasome core complex, integral component of membrane, DNA binding, threonine-type endopeptidase activity, serine-type peptidase activity, zinc ion binding, regulation of transcription, DNA-templated, proteolysis involved in cellular protein catabolic process |
| GRMZM2G073535 | Translation initiation factor activity, translational initiation, regulation of translation |
| GRMZM5G802338 | Translation initiation factor activity, translational initiation |
| GRMZM2G057186 | GDP-D-glucose phosphorylase activity, metabolic process |
| GRMZM2G081928 | Extracellular region, cytoplasmic membrane-bounded vesicle, peroxidase activity, heme binding, metal ion binding, response to oxidative stress, hydrogen peroxide catabolic process, oxidation-reduction process |
| GRMZM2G033884 | Methyltransferase activity, methylation |
| GRMZM2G019746 | Catalytic activity, fatty-acyl-CoA synthase activity, peroxisome, metabolic process, jasmonic acid biosynthetic process |
| GRMZM2G170890 | Mitochondrial fission |
| GRMZM2G012148 | Peptidase activity, proteolysis |

**Supplementary Table 5.** Functional properties of the 13 gene ontology terms shared by all three derived germination traits.

| **GO ID** | **Level** | **Function** | **Type^*^** | **GRMZM2G113158** | **GRMZM2G389768** | **GRMZM2G012148** | **GRMZM2G073535** | **GRMZM5G802338** | **GRMZM2G081928** | **GRMZM2G704005** | **GRMZM2G057186** | **GRMZM2G300994** | **GRMZM2G462797** | **GRMZM2G033884** | **GRMZM2G019746** | **GRMZM2G178486** | **GRMZM5G806387** | **GRMZM2G170890** | **Total** |
| --- | --- | --- | --- | --- | --- | --- | --- | --- | --- | --- | --- | --- | --- | --- | --- | --- | --- | --- | --- |
| **GO:0003674** | 1 | Molecular function | F | √ | √ | √ | √ | √ | √ | √ | √ | √ | √ | √ | √ | √ |  |  | 13 |
| **GO:0005575** | 1 | Cellular component | C | √ | √ |  |  |  | √ |  |  |  | √ |  |  |  | √ |  | 5 |
| **GO:0008150** | 1 | Biological process | P | √ | √ | √ | √ | √ | √ | √ | √ | √ | √ | √ | √ |  |  | √ | 13 |
| **GO:0003824** | 2 | Catalytic activity | F | √ | √ | √ |  |  | √ | √ | √ | √ |  | √ | √ |  |  |  | 9 |
| **GO:0005488** | 2 | Binding | F | √ | √ |  | √ | √ | √ |  |  |  | √ |  |  | √ |  |  | 7 |
| **GO:0008152** | 2 | Metabolic process | P | √ | √ | √ | √ | √ | √ | √ | √ | √ | √ | √ | √ |  |  |  | 12 |
| **GO:0016020** | 2 | Membrane | C | √ | √ |  |  |  |  |  |  |  |  |  |  |  | √ |  | 3 |
| **GO:0016740** | 3 | Transferase activity | F | √ |  |  |  |  |  |  | √ | √ |  | √ |  |  |  |  | 4 |
| **GO:0043167** | 3 | Ion binding | F | √ | √ |  |  |  | √ |  |  |  |  |  |  | √ |  |  | 4 |
| **GO:0044238** | 3 | Primary metabolic process | P | √ | √ | √ | √ | √ |  | √ |  |  |  |  |  |  |  |  | 6 |
| **GO:0071704** | 3 | Organic substance metabolic process | P | √ | √ | √ | √ | √ |  | √ |  |  |  |  |  |  |  |  | 6 |
| **GO:0043170** | 4 | Macromolecule metabolic process | P | √ | √ | √ | √ | √ |  |  |  |  |  |  |  |  |  |  | 5 |
| **GO:0019538** | 5 | Protein metabolic process | P | √ | √ | √ | √ | √ |  |  |  |  |  |  |  |  |  |  | 5 |
| **Frequency** |  |  |  | 13 | 12 | 8 | 8 | 8 | 7 | 6 | 5 | 5 | 5 | 5 | 4 | 3 | 2 | 1 | 92 |

**Supplementary Figure**


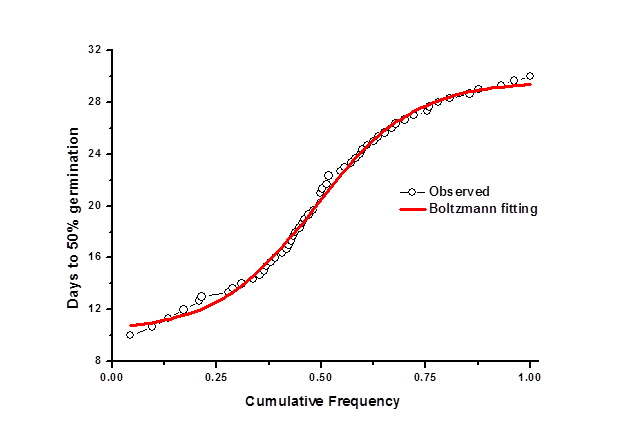


**Supplementary Figure 1. Observed days to 50% germination and Boltzmann fitting curve.** Germination was defined as root emergence from seed. Germination rates were observed daily under chilling conditions (8℃). The Boltzmann growth curve was fitted to the observed days to 50% germination over corresponding cumulative frequency using software OriginPro 8 (http://www.originlab.com). The fitted and observed curves matched well, with an adjusted R square of 99.56%.


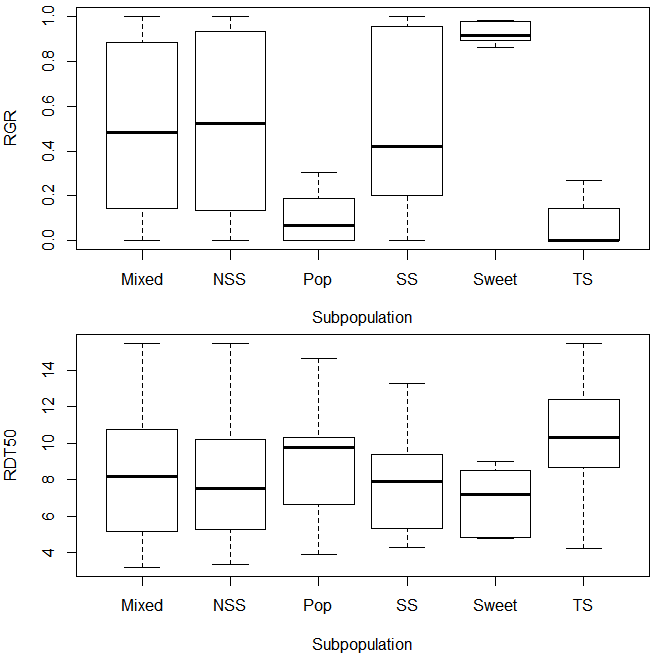


**Supplementary Figure 2. The distribution of germination traits by subpopulations. Boxplots are used to illustrate the distributions within subpopulations under chilling conditions for RGR (the top panel) and RDT50 (the bottom panel).** The subpopulations are Non-Stiff Stalk (NSS), Stiff Stalk (SS), Tropical/Subtropical (TS), Popcorn (Pop), Sweet corn (Sweet) and the rest (Mixed) based on Flint-Garcia et al. (2005).


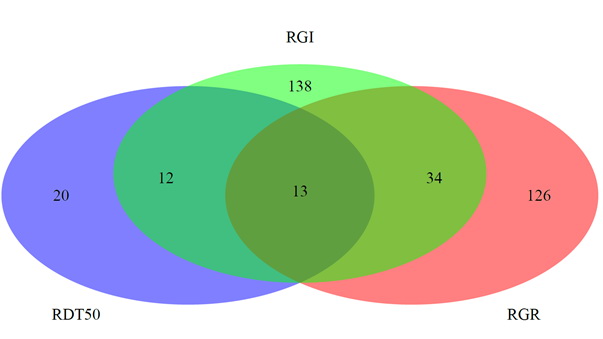


**Supplementary Figure 3. Numbers of gene ontology (GO) terms shared by 18 candidate genes associated with three derived germination traits.** The three traits (RDT50, RGI, and RGR) were derived as relative values by dividing the germination trait values (DT50, GI, and GR) under chilling conditions by their corresponding trait values under normal conditions. Germination was defined as root emergence from seed. In total, we found 343 GO terms characterizing the 18 candidate genes associated with the three traits. Most of the GO terms were specific to each trait. Only 55.6%, 29.9%, and 27.2% of the GO terms for RDT50, RGI, and RGR, respectively, were shared among each other. Although RGR did not share any associated SNPs with RDT50 and RGI, we still found 13 GO terms shared by all three traits.


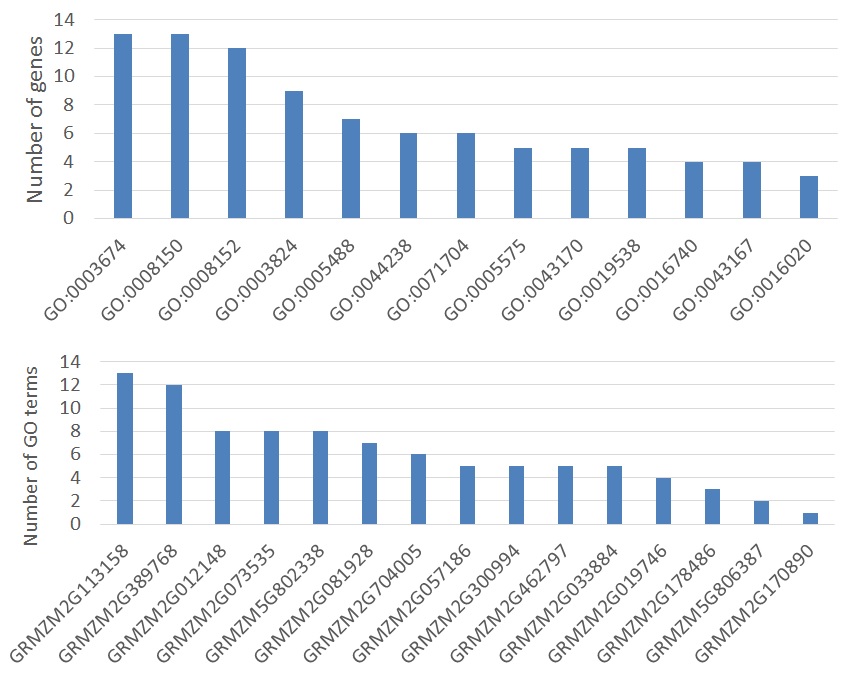


**Supplementary Figure 4. Distribution of genes and GO terms.** Distribution of number of genes per GO terms is illustrated at the top panel and number of GO terms per gene is illustrated at the bottom panel.


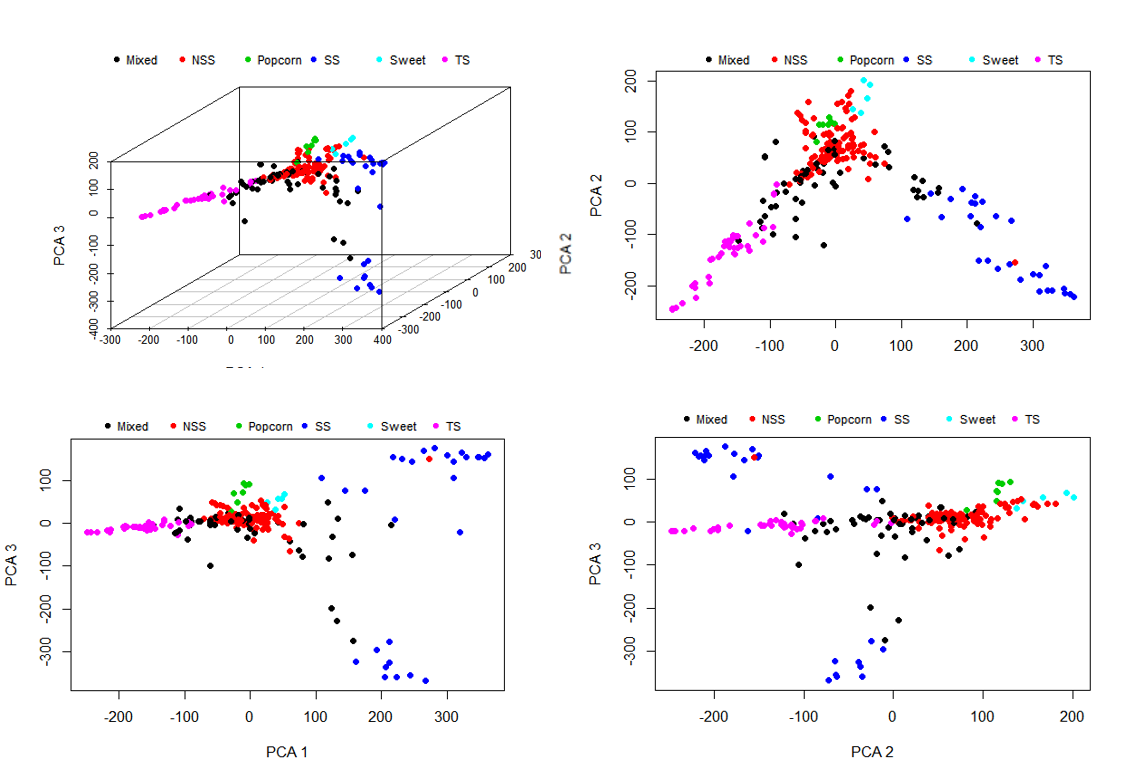


**Supplementary Figure 5. Population structure of the 241 inbred lines.** The population structure is represented by the first three principal components (PC1, PC2 and PC3) in three dimensions, and their pairwise relationship in two dimensions. Inbred lines are colored based on corresponding subpopulation. The subpopulations are Non-Stiff Stalk (NSS), Stiff Stalk (SS), Tropical/Subtropical (TS), Popcorn (Pop), Sweet corn (Sweet) and the rest (Mixed) based on Flint-Garcia et al. (2005).
